# Yes-associated protein 1 promotes papillary thyroid cancer cell proliferation by activating the ERK/MAPK signaling pathway

**DOI:** 10.18632/oncotarget.14319

**Published:** 2016-12-28

**Authors:** Tian Liao, Duo Wen, Ben Ma, Jia-Qian Hu, Ning Qu, Rong-Liang Shi, Liang Liu, Qing Guan, Duan-Shu Li, Qing-Hai Ji

**Affiliations:** ^1^ Department of Head and Neck Surgery, Fudan University Shanghai Cancer Center, Department of Oncology, Shanghai Medical College, Fudan University, Shanghai 200032, China; ^2^ Department of Breast Surgery, Breast Cancer Institute, Shanghai Cancer Center, Department of Oncology, Shanghai Medical College, Fudan University, Shanghai, 200032, China

**Keywords:** papillary thyroid cancer, YAP1, proliferation, signaling pathway

## Abstract

Yes-associated protein 1 (YAP1) stimulates cell proliferation, epithelial-to-mesenchymal transition, invasion and metastasis in several cancers. Here, we investigated the involvement of YAP1 in papillary thyroid carcinoma (PTC) by assessing *YAP1* mRNA and protein levels in PTC tissues and matched normal thyroid epithelial tissues from 50 patients. *YAP1* mRNA and protein levels were higher in PTC tumor tissues than in control tissues, and correlated positively with the levels of proliferation-related genes (*KI67* and *c-MYC*). We also used lentiviral vectors to overexpress or silence *YAP1* expression in the K1 PTC cell line so that we could investigate the effects of YAP1 on cancer cell proliferation. *YAP1* overexpression enhanced PTC cell proliferation by activating ERK1/2 and AKT, and these effects were impaired by treating the cells with the MEK inhibitor U0126 or the AKT inhibitor GSK690693. Finally, *YAP1* overexpression dramatically induced growth of tumors from PTC cells in a xenograft mouse model. These results suggest that YAP1 enhances cell proliferation in PTC, and thus may be a promising target in the treatment of PTC.

## INTRODUCTION

The incidence of thyroid carcinoma is increasing by 4% per year, and papillary thyroid carcinoma (PTC) accounts for approximately 80% of thyroid carcinoma cases [1]. The biological behavior of PTC varies widely: some tumors exhibit little or no invasion, while others become aggressive, metastasize and cause death. Like the majority of malignancies, thyroid carcinomas are usually associated with specific genetic abnormalities that cause aberrant cell proliferation [2]. Thus, studying the molecular pathogenesis of thyroid carcinoma is an important primary step in the search for therapeutic targets and more effective diagnostic and prognostic markers.

Knowledge of signaling networks has become increasingly important for our understanding of cell proliferation. Yes-associated protein 1 (YAP1) is a transcriptional regulator of the Hippo signaling pathway, and is known to play a role in development, growth, repair and homeostasis [3]. YAP1 is also known to promote cell proliferation, epithelial-to-mesenchymal transition, invasion and metastasis [4]. Multiple studies have demonstrated that *YAP1* is overexpressed in various types of solid tumors, including breast [5], hepatocellular [6] tumors. In these cancers, high *YAP1* expression is associated with tumor initiation, invasion and metastasis, suggesting that YAP1 promotes tumorigenesis.

However, the involvement of YAP1 in PTC remains largely unexplored to date. In this study, we hypothesized that YAP1 activation is critical for the maintenance and proliferation of PTC. We therefore tested the function of YAP1 directly in a PTC cell line and tumor tissues. Here, we report that YAP1 induces the proliferation of PTC cells *in vitro* and *in vivo* by stimulating ERK/MAPK signaling.

## RESULTS

### *YAP1* and proliferation-related genes are upregulated in PTC cells

**Figure 1:**
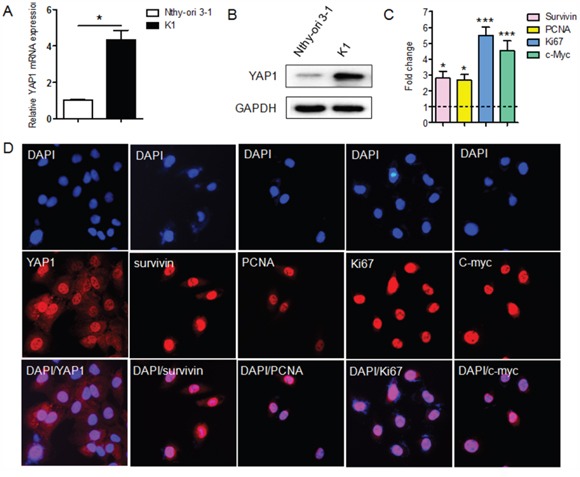
The expression of YAP1 and proliferation-related genes in the PTC cell line, K1 **A** and **B**. Both mRNA (A) and protein levels (B) of *YAP1* were greater in PTC cells (K1) than in normal human thyroid epithelial cells (Nthy-ori 3-1) (*P<0.05). **C**. Thelevels of proliferation-related genes (*Survivin*, *PCNA*, *KI67* and *c-MYC*) were greater in K1 cells than in Nthy-ori 3-1 cells, as determined by qPCR. The results are presented as fold-changes in the PTC cell line relative to the Nthy-ori-3 cell line (*P<0.05, ***P<0.001). **D**. YAP1 was localized mostly in the nuclei of K1 cells, as were the proliferation-related proteins (Original magnification ×200). qPCR results were normalized to *β-actin* mRNA expression. All the experiments were performed in triplicate.

The expression of *YAP1* was evaluated in human normal thyroid epithelial cells (Nthy-ori 3-1) and PTC cells (K1) by qRT-PCR. *YAP1* expression was significantly greater in K1 cells than in Nthy-ori 3-1 cells (Figure 1A), consistent with the results of a prior study [7]. Western blotting analysis revealed that YAP1 protein expression was upregulated in K1 cells (Figure 1B), further confirming the qRT-PCR data. Next, we evaluated the expression of proliferation-related genes (*Survivin*, *PCNA*, *KI67* and *c-MYC*) in Nthy-ori 3-1 and K1 cells by qRT-PCR. As shown in Figure 1C, the levels of proliferation-related genes were higher in K1 cells than in Nthy-ori 3-1 cells. In addition, immunofluorescent staining results revealed that YAP1 was localized mostly in the nuclei of K1 cells, as were the proliferation-related genes (Figure 1D).

### *YAP1* overexpression and knockdown regulate the proliferation of PTC cells *in vitro*

**Figure 2:**
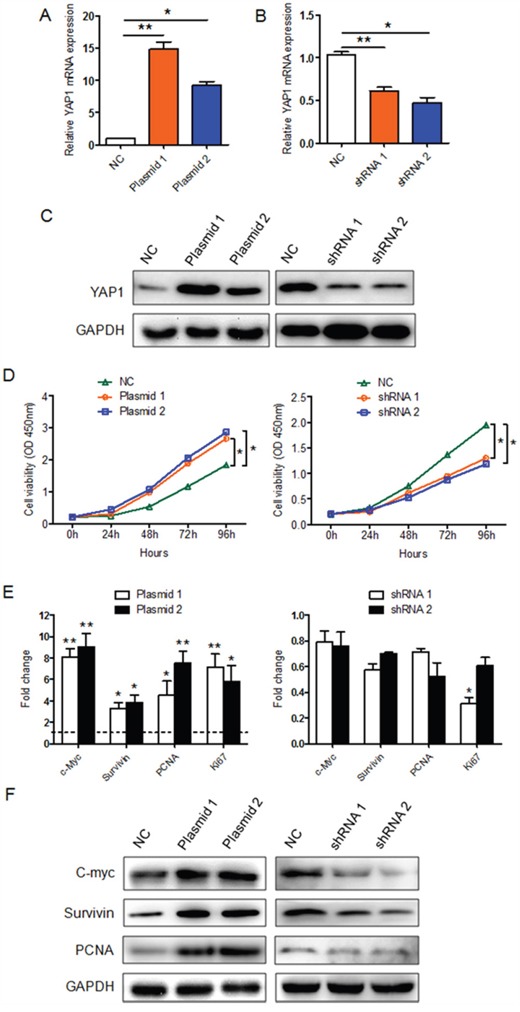
The effect of YAP1 on K1 cell proliferation *in vitro* **A-C**. *YAP1*-overexpressing K1 cells and YAP1-knockdown K1 cells were generated through transfection of K1 cells with *YAP1* plasmids or lentivirus-*YAP1*, respectively. *YAP1* expression in the infected cells (K1-*YAP1* plasmid and K1-shRNA *YAP1*) was confirmed by qPCR and Western blotting (NC: negative control). qPCR results were normalized to *β-actin* mRNA expression. **D**. CCK-8 cell proliferation assays were performed on *YAP1*-overexpressing K1 cells (left part) or YAP1-knockdown K1 cells (right part). The results are presented as the mean ± SEM from three independent experiments. **E** and **F**. The levels of proliferation-related genes in *YAP1*-overexpressing K1 cells (left part) or YAP1-knockdown K1 cells (right part) were evaluated by qPCR (E) and Western blotting (F). The qPCR results are presented as fold-changes in infected K1 cells relative to non-infected K1 cells (negative control) (*P<0.05, **P<0.01, ***P<0.001).

To determine whether YAP1 is necessary for the proliferation of PTC cells, we first knocked down YAP1 in K1 cells using one of two lentiviral constructs (lenti-shRNA 1 and 2). We also overexpressed *YAP1* in PTC cells by transfecting K1 cells with one of two *YAP1* plasmids (*YAP1* plasmids 1 and 2). The silencing or ectopic expression of *YAP1* was confirmed by qRT-PCR (Figure 2A and 2B) and Western blotting (Figure 2C). Cell Counting Kit-8 (CCK8) assays demonstrated that the overexpression of *YAP1* (especially with the *YAP1* plasmid 1) enhanced the viability of PTC cells, while the silencing of YAP1 reduced the viability of K1 cells (Figure 2D). In addition, the levels of proliferation-related genes were elevated in *YAP1*-overexpressing K1 cells and reduced in YAP1-knockdown K1 cells, at both the mRNA (Figure 2E) and protein levels (Figure 2F). Thus, YAP1 may promote the proliferation of human PTC cells.

### YAP1 promotes the proliferation of PTC cells in a xenograft mouse model

**Figure 3:**
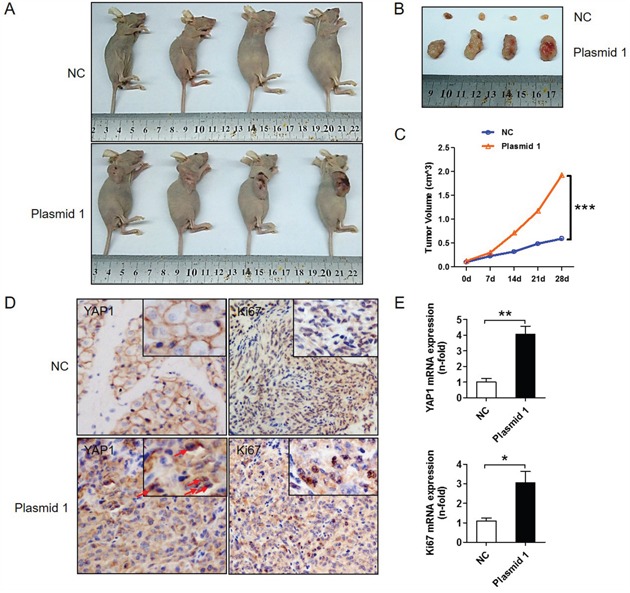
YAP1 promotes tumor growth *in vivo* *YAP1*-overexpressing (K1-plasmid 1 *YAP1*) cells and control cells (K1-NC) were injected into BALB/c nude mice. **A** and **B**. Four representative tumors from each group (n=4) are shown. **C**. Volume comparison for tumors from the K1-plasmid 1 *YAP1* group and the K1-NC group. **D**. IHC analysis of YAP1 (nucleus expression marked by red arrows) and KI67 expression in formalin-fixed, paraffin-embedded tumors from the K1-plasmid 1 *YAP1* group and the K1-NC group (Original magnification ×200). **E**. *YAP1* and *KI67* levels in tumors from the K1-plasmid 1 *YAP1* group and the K1-NC group were determined by qPCR and normalized to *β-actin* mRNA expression (*P<0.05, **P<0.01, ***P<0.001).

A xenograft mouse model was used to determine the effect of YAP1 on PTC cell proliferation *in vivo*. *YAP1*-overexpressing K1 cells or control cells (K1-NC) were subcutaneously injected into nude mice. As shown in Figure 3A and 3B, tumor growth increased dramatically in mice injected with *YAP1*-overexpressing K1 cells compared with those injected with control cells. After 4 weeks, each of the four mice injected with K1-NC cells developed a small tumor (tumor volume < 0.5 cm^3^), whereas each of the four mice injected with *YAP1*-overexpressing K1 cells grew a large tumor (tumor volume > 1.0 cm^3^) (Figure 3C), indicating that the latter cells were more tumorigenic than the former. In addition, the protein and mRNA levels of *YAP1* and *KI67* were significantly greater in tumors generated from *YAP1*-overexpressing K1 cells than in those from control cells (Figure 3D and 3E). These *in vivo* data are consistent with the above *in vitro* results, suggesting that YAP1 may be a vital promoter of the tumorigenesis and maintenance of human PTC.

### YAP1 promotes PTC cell proliferation by activating the ERK/MAPK signaling pathway

**Figure 4:**
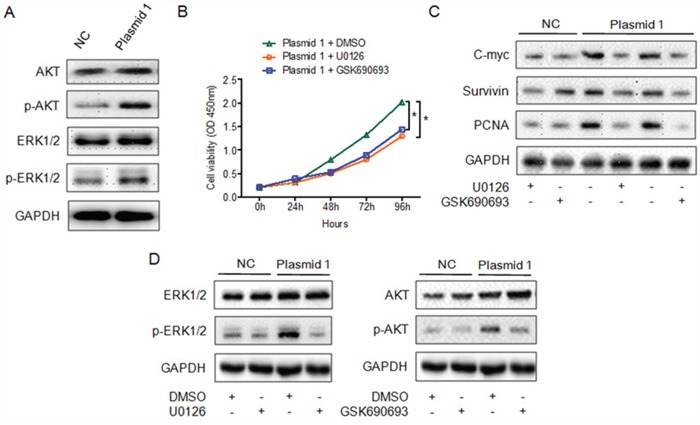
YAP1 enhances the proliferation of PTC cells by inducing the ERK/MAPK signaling pathway **A**. Analysis of total and phosphorylated ERK1/2 and AKT levels in K1-plasmid 1 *YAP1* cells. **B**. CCK-8 cell proliferation assay demonstrating inhibition of the proliferation of K1-plasmid 1 *YAP1* cells treated with U0126 or GSK690693 (*P<0.05). **C** and **D**. Treatment of K1-plasmid 1 *YAP1* cells with U0126 or GSK690693 suppressed the expression of proliferation-related genes (C) and phosphorylated ERK1/2 or AKT (D).

To investigate whether MAPK signaling participates in the YAP1-induced proliferation of PTC cells, we assessed the expression of total and phosphorylated AKT and ERK1/2 in *YAP1*-overexpressing K1 cells and control cells. AKT and ERK1/2 phosphorylation were greater in *YAP1*-overexpressing cells than in control cells, while the total AKT and ERK1/2 levels remained unchanged (Figure 4A). Moreover, treatment with either U0126 (a MEK inhibitor) or GSK690693 (an AKT inhibitor) significantly reduced the viability of *YAP1*-overexpressing K1 cells (Figure 4B). The levels of proliferation-related genes were lower in *YAP1*-overexpressing K1 cells than in control cells following treatment with U0126 or GSK690693 (Figure 4C). As expected, the levels of phosphorylated ERK1/2 and AKT, but not total ERK1/2 and AKT, were reduced following exposure of cells to either U0126 or GSK690693 (Figure 4D). Thus, YAP1 promoted the proliferation of PTC cells by inducing ERK/AKT signaling.

### *YAP1* expression is elevated in human PTC tissues and correlates positively with *KI67* and *c-MYC* expression

**Figure 5:**
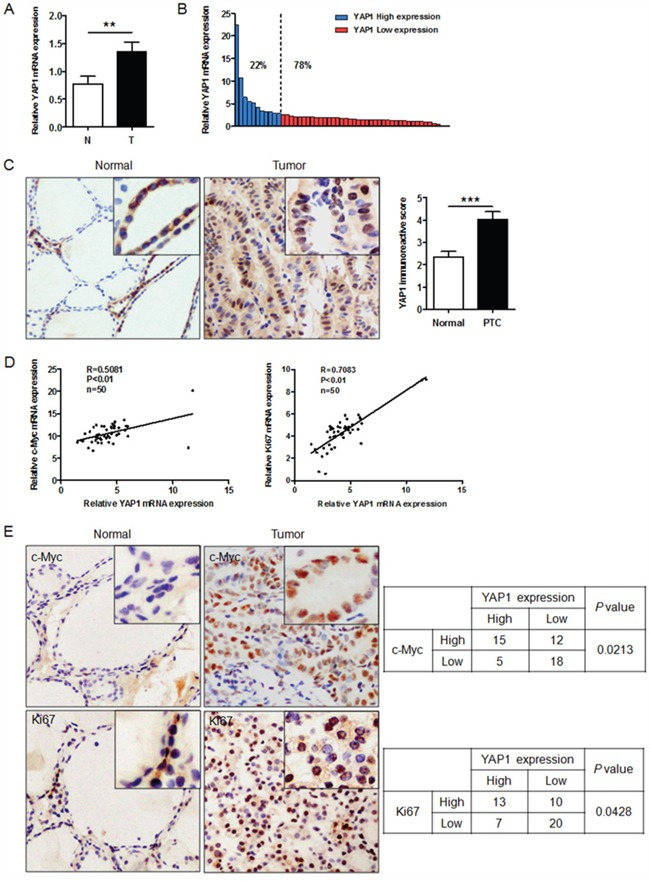
YAP1 expression is elevated in human PTC tissues and correlates positively with KI67 and c-MYC expression **A**. Relative levels of *YAP1* in PTC (T) and paired adjacent normal (N) tissues (n=50), examined by qPCR and normalized to *β-actin* mRNA levels (**P<0.01). **B**. *YAP1* levels in PTC tissues were classified as high (22% of samples) or low (78% of samples) according to the average *YAP1* level. **C**. YAP1 expression was higher in PTC tissues than in adjacent normal thyroid tissues (Original magnification ×200). **D** and **E**. Both mRNA (D) and protein levels (E) of *YAP1* correlated positively with *KI67* and *c-MYC* mRNA and protein levels (Original magnification ×200).

To further validate the above data, we performed qRT-PCR to examine the mRNA levels of *YAP1* in 50 cases of human PTC and adjacent non-malignant thyroid tissues. *YAP1* expression was greater in PTC tissues than in adjacent non-malignant thyroid tissues (Figure 5A). Based on their average relative *YAP1* levels, the 50 PTC tissues were classified into the high- and low-*YAP1*-expression groups. Among these PTC samples, 11 of 50 (22%) expressed high levels of *YAP1*, whereas 39 of 50 (78%) expressed low levels (Figure 5B). Next, we detected YAP1 protein levels in PTC tissues by immunohistochemistry (IHC). As shown in Figure 5C, YAP1 was weakly expressed or not expressed in non-malignant thyroid tissues, but was highly expressed in the nuclei of tumor specimens, in line with a previous observation [7]. Correlation analysis indicated that the immunostaining intensity of YAP1 was significantly positively associated with those of KI67 (*P*<0.05) and c-MYC (*P*<0.05) (Figure 5E). Furthermore, mRNA data also confirmed the relationship of *YAP1* levels with *KI67* and *c-MYC* levels in PTC tissues (Figure 5D). These findings provided additional support to the notion that YAP1 promotes the proliferation of PTC cells.

## DISCUSSION

In the present study, we found that *YAP1* levels were elevated in human PTC tissues and a PTC cell line. A positive correlation was observed between the expression of *YAP1* and the expression of proliferation-related genes (*KI67* and *c-MYC*) in tumor specimens. In gain-of-function analyses, overexpression of *YAP1* augmented the proliferation of PTC cells and the expression of proliferation-related genes (*Survivin*, *PCNA*, *KI67* and *c-MYC*) *in vitro* and tumorigenicity *in vivo*. Withdrawal of YAP1 had the opposite effects on human PTC cells. Furthermore, we discovered that YAP1 promoted PTC cell proliferation by activating the ERK/MAPK signaling pathway, while pharmacologic inhibition of MEK or AKT abolished these effects. Thus, our results manifested that YAP1 may stimulate PTC pathogenesis by boosting PTC cell proliferation.

The importance of the Hippo signaling pathway in the regulation of organ size and cellular proliferation is well recognized. YAP1, an effector of the Hippo signaling pathway, has been reported to be an oncogene that stimulates proliferation, tumorigenicity and metastasis in several tumor types [8–12]. However, the effect of YAP1 on the proliferation of PTC cells had not yet been demonstrated. Our investigation has provided evidence that *YAP1* expression is elevated in human PTC tumor tissues and K1 cells. As expected, YAP1, as a transcriptional coactivator, was mostly retained in the nuclei of K1 cells. Lee et al. also observed the upregulation and nuclear localization of YAP1 in K1 cells [7], but they did not determine the biological function of YAP1 in these cells. In the current study, we demonstrated for the first time that YAP1 promotes K1 cell proliferation *in vitro* and tumorigenicity *in vivo*. The positive relationship of *YAP1* expression with *KI67* and *c-MYC* expression in PTC tissues further illustrates the importance of YAP1 for PTC cell proliferation. These data suggest that YAP1 might be an oncogene in PTC tumorigenesis.

The MAPK signaling pathway is reported to induce cell proliferation in multiple tumor types [13–15], including thyroid cancer. Li et al. found that YAP1 accelerated tumor growth in the human gallbladder by activating the AXL/MAPK pathway [16]. Nevertheless, it was not clear whether YAP1 could also stimulate MAPK signaling in PTC. Our data indicated that activation of YAP1 increased the phosphorylation of ERK1/2 and AKT *in vitro*. Thus, nuclear YAP1 may induce ERK1/2 and AKT phosphorylation to activate downstream signaling in the MAPK pathway. Furthermore, exposure to the MEK inhibitor U0126 or the AKT inhibitor GSK690693 partly blocked these MAPK-induced effects. Hence, ERK/MAPK and AKT signaling may participate in the YAP1-induced proliferation of PTC in a variety of *in vitro* contexts. Given the induction of the ERK/MAPK signaling pathway in *YAP1*-overexpressing PTC cells, YAP1 may be a viable therapeutic target for the treatment of PTC.

In the present study, we have declared that YAP1 may act as a tumor inducer by stimulating the ERK/MAPK signaling pathway in PTC. Nonetheless, there are some limitations to this preclinical pilot study. First, there was no correlation between YAP1 mRNA expression and patient clinical characteristics (data not shown). It may be that 50 tumor specimens were too few to manifest a clinical correlation. Therefore, a large number of subjects should be examined to solidify these data in the future. However, we have provided evidence that elevated *YAP1* expression activates the ERK/MAPK pathway to promote cell proliferation in PTC. Further investigation of the underlying mechanism is still needed to determine how YAP1 triggers the ERK/MAPK pathway to induce proliferation in PTC.

In conclusion, we have demonstrated for the first time that YAP1 induces cell proliferation and tumorigenicity in PTC cells, partly by activating the ERK/MAPK signaling pathway, and that *YAP1* expression is upregulated in human PTC tissues. These preclinical findings suggest that the inhibition of *YAP1*, alone or in combination with other potential markers, may effectively combat PTC.

## MATERIALS AND METHODS

### Tissue specimens

A total of 50 thyroid cancer samples were obtained from patients who had undergone thyroid cancer surgery between 2012 and 2015 at Shanghai Cancer Center, Fudan University. Tissue specimens were frozen in liquid nitrogen immediately after surgical resection and stored at -80°C until use. Final histological classification was determined from paraffin-embedded sections. For the use of the clinical materials for research purposes, the Institutional Research Ethics Committee approved the study, and prior patient consent was required.

### Cell lines and transfection

Nthy-ori 3-1 (purchased from Sigma-Aldrich company) and K1 (purchased from University of Colorado Cancer Center Cell Bank) cells were grown in RPMI1640 medium supplemented with 10% FBS (Invitrogen, Carlsbad, CA, USA) and 1% penicillin-streptomycin (10,000 units penicillin and 10 mg streptomycin per mL in 0.9% NaCl, Sigma-Aldrich) at 37°C with 5% CO_2_. Knockdown or overexpression of *YAP1* in K1 cells was accomplished by lentiviral infection: HEK-293T cells were co-transfected with pLKO.1-YAP1 or pCDH3.1-YAP1 constructs and packaging plasmids. The medium containing viruses released from the HEK-293T cells was used to infect the K1 cells and thus establish K1-shRNA *YAP1* cells or K1-plasmid *YAP1* cells. Then, selection was performed with 2 μg/mL puromycin (Sigma-Aldrich).

### Drug treatments

K1-plasmid *YAP1* cells were grown as described above until they became 40% confluent. Then, the media were replaced with RPMI1640 containing vehicle (0.1% (v/v) dimethyl sulphoxide), U0126 (10 μM) (Promega, Madison, WI) or GSK690693 (10 μM) (Sigma Chemical Co., St. Louis, MO). Samples were collected at different time intervals as indicated for each experiment.

### RNA extraction and RT-PCR

Total RNA was extracted from cultured cells with TRIzol Reagent (Invitrogen, Inc.). Then, 1 μg of total RNA was used for first-strand cDNA synthesis by means of a PrimeScript™ RT reagent kit (Takara Bio, Inc., Japan). Real-time PCR was performed in triplicate by the SYBR Green PCR method, with a SYBR Premix Ex Taq™ kit (Takara Bio, Inc., Japan) in accordance with the manufacturer's instructions. The primers for the genes of interest were synthesized by Sangon Company (Sangon Biotech Co., Ltd., Shanghai, China) (Table 1). *β-actin* was used as an internal control for mRNA assays. The cycle threshold (Ct) values were analyzed through the comparative Ct (2^-ΔΔCt^) method. The expression of each target was normalized to that of the endogenous reference and expressed relative to that of the appropriate control.

**Table 1:** qRT-PCR primers of target genes

Target genes	Forward sequence (5'to3')	Reverse sequence (5'to3')
YAP1	TAGCCCTGCGTAGCCAGTTA	TCATGCTTAGTCCACTGTCTGT
c-Myc	GGAGGCTATTCTGCCCATTT	CGAGGTCATAGTTCCTTGTTGGT
survivin	CACTTTCTTCGCAGTTTCCT	GACCACCGCATCTCTACATTC
PCNA	AAACTAGCTAGACTTTCCTC	TCACGCCCATGGCC AGGTTG
Ki67	*ACCCTGCGACTCTCCACAGT*	*GCTCCTCTGTACGTCCCTTTT*
β-actin	TGACGTGGACATCCGCAAAG	CTGGAAGGTGGACAGCGAGG

### Western blotting

Lysates were obtained from 1 × 10^6^ cultured cells with a mixture of Proteo^JET^ Mammalian Cell Lysis Reagent (Fermentas, Inc.), phenylmethanesulfonyl fluoride (Roche, Inc.) and PhosSTOP (Roche, Inc.). About 20 μg protein was extracted from each sample and separated by 10% sodium dodecyl sulfate polyacrylamide gel electrophoresis (SDS-PAGE). After the membrane was blocked in 5% nonfat milk, the protein of interest was probed with the appropriate antibody: human YAP1 (1:1000; Cell Signaling Technology), c-MYC (1:1000; abcam), Survivin (1:1000; abcam), KI67 (1:1000; Cell Signaling Technology), PCNA (1:1000; Cell Signaling Technology), ERK1/2 (1:1000; Cell Signaling Technology), p-ERK1/2 (1:1000; Cell Signaling Technology), AKT (1:1000; Cell Signaling Technology), p-AKT (1:1000; Cell Signaling Technology), or GAPDH (1:5000; Abcam). Then, the membrane was incubated with goat anti-rabbit or anti-mouse IgG (1:5000 for both; Jackson ImmunoResearch Laboratories) and treated with enhanced chemiluminescence reagents (Thermo Fisher Scientific). The bands were visualized with 1-stepTM NBT/BCIP reagents (Thermo Fisher Scientific, Rockford, IL, USA) and detected with Alpha Imager (Alpha Innotech, San Leandro, CA, USA).

### Cellular viability assays

Cell proliferation was determined with a CCK-8 assay according to the manufacturer's protocol. Briefly, cells were seeded into 96-well plates at 4×10^3^ cells/well. An aliquot of 10 μL CCK-8 solution was added to each well, and the plate was incubated for 4 h at 37°C. At the indicated time points, the absorbance at 450 nm was measured with a spectrophotometer. For each group, data from five wells were pooled.

### Immunofluorescence

Cells were cultured as described above. Then, cells were washed twice with cold PBS and fixed in 4% paraformaldehyde for 20 min at 4°C. After three PBS washes, cells were permeabilized with 0.1% Triton X–100 for 5 min and incubated in PBS containing 1% BSA for 30 min, followed by overnight incubation at 4°C with primary antibodies. After the cells were successively washed, an Alexa Fluor 594-conjugated secondary antibody (1:500; Invitrogen) was added for a 1-h incubation at room temperature. Then, the cells were washed with PBS, and nuclei were labeled with 4’,6-diamidino-2-phenylindole (DAPI) for 2 min. Cover slips were then mounted with anti-fading solution and observed under a fluorescence microscope (Olympus X–50).

### Immunohistochemical staining

IHC was carried out according to the manufacturer's protocol. Briefly, formalin-fixed and paraffin-embedded tissue sections were deparaffinized in xylene and hydrated with decreasing concentrations of ethanol before being placed in a blocking solution to inhibit endogenous peroxidase activity. The slides were incubated with primary antibodies at 4°C overnight. A horseradish peroxidase-conjugated rabbit or mouse secondary antibody was added for 60 min at room temperature; then, 3, 3′-diaminobenzidine (DAB) development (DAB Substrate Chromogen System, Dako) and hematoxylin and eosin (H&E) staining were performed according to standard protocols. Slides were fixed and images were obtained through an Olympus IX71 inverted microscope with a DP2-BSW Olympus image acquisition software system. The results were confirmed by two experienced pathologists who were blinded to the clinicopathologic data of the patients. The staining results were calculated on the basis of the percentage of tumor cell nuclei stained (0, no staining; 1, ≤10%; 2, 10%-50%; and 3, > 50%) and the staining intensity (0, negative; 1, weak; 2, moderate; and 3, strong). These scores were multiplied, such that an overall score of 1-5 designated low expression, and an overall score of 6-9 designated high expression for both YAP1 and the proliferation-related proteins in the 50 PTC tissue samples [17].

### Animal experiments

Five-week-old BALB/c nude mice were obtained from the Shanghai experimental animal center (Shanghai, China). Briefly, 1×10^7^ cells were subcutaneously injected into the right back skin area of the mice. Tumor size was measured with a vernier caliper twice per week. After 28 days, mice were sacrificed, and tumor tissues were collected, photographed and examined. Paraffin-embedded tissues were sectioned for IHC analysis. Part of the tumor tissue was frozen in liquid nitrogen for subsequent experiments. The animal experiments were pre-approved by the Shanghai Cancer Center, Fudan University, and all procedures were performed in accordance with the National Institutes of Health Guide for the Care and Use of Laboratory Animals.

### Statistics

Statistical analyses were performed with GraphPad Prism 5.1. For comparisons among the groups, a Student's *t* test, one-way ANOVA, and a Chi-Square test were performed, and *P*<0.05 was defined as statistically significant. The data and error bars represent the mean ± SEM. Each experiment was repeated at least three times.
